# Improved Mechanical and Sound Absorption Properties of Open Cell Silicone Rubber Foam with NaCl as the Pore-Forming Agent

**DOI:** 10.3390/ma14010195

**Published:** 2021-01-03

**Authors:** Longgui Peng, Lei Lei, Yongqiang Liu, Lifei Du

**Affiliations:** College of Materials Science and Engineering, Xi’an University of Science and Technology, Xi’an 710054, China; leilei_xust@163.com (L.L.); liuyongqiang_1997@foxmail.com (Y.L.)

**Keywords:** silicone rubber, open cell foam, sound absorption properties, mechanical properties

## Abstract

Porous materials hold great potential in the field of sound absorption, but the most abundantly used materials, such as Polyurethane (PU) foam and polyvinyl chloride (PVC) foam, would inevitably bring environmental harms during fabrication. In this study, the nontoxic addition-molded room temperature vulcanized silicone rubber is chosen as the matrix, and NaCl particles are chosen as the pore forming agent to prepare open cell foams via the dissolve-separating foaming method. The effect of different amounts of NaCl (0–100 phr) on the cell structure, mechanical and sound absorption properties is investigated and analyzed. The results indicate that the cell structure could be tailored via changing the addition amount of NaCl, and open cell silicon rubber foams could be achieved with more than 20 phr NaCl addition. Open cell silicon foams show the most effective sound absorption for sound waves in middle frequency (1000–2000 Hz), which should be attributed to the improved impedance matching caused by the open cell structures. Additionally, the mechanical properties, including hardness, tensile strength and corresponding elastic properties, gradually decay to a steady value with the increasing addition amount of NaCl. Therefore, open cell silicone rubber foams are capable of sound absorption in middle frequency.

## 1. Introduction

With the rapid development of modern industry, transportation and urban construction, noise pollution has become critical issues in modern society [1]. Noise and vibration will not only reduce industrial production efficiencies, but also be harmful to workers in industrial and noisy environments [2]. Therefore, reducing the harm caused by noise and vibration has drawn increasing attention in the fields of scientific research, industrial and so on [3,4,5]. According to the characteristics of noise propagation, noise can be controlled in terms of noise source, noise propagation path, and noise receiver. Among them, controlling noise in the propagation path is the simplest and most effective way. At present, two methods are widely used in noise reduction: active noise reduction and passive noise reduction. Active noise reduction consists of generating sound by attaching secondary sound sources, which has the same amplitude and frequency, but the opposite phase as the main noise to achieve the purpose of sound elimination. Passive noise reduction is used to implement sound insulation and sound absorption technology. Among them, foams are the widely designed and investigated materials to realize effective sound absorption in broadband frequency [6,7,8].

Foam-like sound-absorbing material generally has sponge-like or foam-like structures inside the matrix, which is effective for sound absorption in the frequency range of 500–2000 Hz. Metal, glass and plastic could all be used make foam-like sound-absorbing materials. Metal foams inherit intrinsic properties from the metallic matrix, including great stiffness and strength, good refractoriness, good temperature change adaptability, low moisture absorption, and excellent impact energy absorption, etc. Metal foams with designed cavities are widely used for noise and vibration control in aircraft, locomotives, automobiles, machines, and buildings [9,10,11]. Glass Foam is an inorganic non-metallic material made of broken glass slag as a raw material, and other various additives such as foaming agents, aids, modifiers, etc. Foam glass used for sound absorption and noise reduction always has a closed-cell structure, light weight, non-combustible, good corrosion resistance and weather resistance, and is widely used in sound absorption and noise reduction in special environments due to the resonance effect. Plastic foams are a polymer material with solid plastic as the matrix and a large number of gas distributed micropores inside. Compared to pure plastic, plastic foams have low density, light weight, good impact resistance, low thermal conductivity, good heat insulation, and excellent sound absorption performance, and they are widely used in industries due to their low cost and ease of syntheses [4,6,12,13]. Polyurethane (PU) foam and polyvinyl chloride (PVC) foam are currently the two most common materials used for indoor sound absorption and noise reduction. However, these two types of foam materials would inevitably bring environmental harm during the production process and consequently have an effect on human health [4,14].

Addition-molded room temperature vulcanized silicone rubber is an ideal non-toxic and harmless functional material, meeting the requirement of green environmental protection [15,16,17]. By introducing chemical foaming agents, physical foaming agents or pore forms into the matrix, cell structures inside the silicone rubber foams can be tailored via different additive contents and the vulcanization process. However, silicone rubber foams prepared by traditional chemical foaming methods mainly form the closed-cell structure, which has poor noise absorption efficiency in a narrow frequency band, limiting its wide application for sound absorption [15,16,18]. It has been well established that open cell porous materials exhibit higher efficiency than close cell porous materials in absorbing sound energy due to optimal air-flow resistance and tortuosity by passing through longer path, which is analogous to increasing the thickness of the foam. The dissolve-separating foaming method is an effective way to prepare high performance silicone foam materials with better compression properties and lower relaxation rates [19]. For silicone rubber foam, the vulcanization and pore formation are carried out step by step, and the dissolved filler is precipitated after the silicone rubber is vulcanized and molded. The prepared foamed silicone rubber generally has high porosity, uniform pore density and controllable cell size. Additionally, the cavity characteristics are highly dependent on the vulcanization process parameters (temperature and time) and the type and amount of the additive agents.

In this study, the vinyl-terminated silicone resin and NaCl are chosen as the matrix and pore-forming agent, respectively. The silicone rubber foams with open cell structures are prepared via the dissolve-separating foaming method, and the effect of NaCl addition on the pore structure, sound absorption performance, and other related properties are studied.

## 2. Materials and Methods

Vinyl terminated silicone resin (50 g), white carbon black (15 g), hydrosilicone oil (1.5 g) were mixed with different amounts (10–100 g) of NaCl (Analytical reagent, Tianjin Kemeiou Chemical Reagent Co. LTD, Tianjin, China) in a precision mill for 20 min. Then, the inhibitor (2-methyl-3-Butyn-2-ol) was added to prevent partial vulcanization by inhibiting the crosslinking process. After the blending system was evenly dispersed, platinum catalyst was added into the mixture. Another 10 min mixing was implemented with a smaller roller pitch to make the platinum catalyst uniformly dispersed. The blend was then put into the mold, rolled evenly and placed to the vacuum drying oven for constant pressure vulcanization (24 N, 150 °C, 20 min). After the colloid is vulcanized and molded, the foam was formed by the dissolve-separating foaming method. The fully vulcanized silicone rubber was immersed in a constant temperature heating magnetic stirrer with boiling water for 6 h and taken out to dry in a vacuum drying oven for 1 h at 100 °C. The NaCl crystals was dissolved into water and was then precipitated out from the inside of the silicone rubber through the surface pores during drying. After repeatedly boiling water bath, drying and flushing, the NaCl inside the silicone rubber was completely dissolved to form an inter-connected porous structure.

The internal microstructure of the samples was analyzed via characterizing the cut side of the sample by the scanning electron microscopy (SEM, JSM-6460LV, Japan Electronics Co. LTD., Beijing, China). The water absorption *W* was calculated from the measured weights of the dried (*G*) and water saturated samples (*B*) via *W* = (*B* − *G*)/*G* × 100% under standard atmospheric pressure. The porosity *p* was calculated from the apparent density of non-densified and silicone rubber sample (*ρ*) and the density of densified and silicone rubber sample (*ρ*_0_ = 1.03 g/cm^3^) via *p* = (*ρ* − *ρ*_0_)/*ρ*_0_ × 100%. The apparent density *ρ* was tested by the sealing wax method according to GB/T 6342-2009. The falling rebound rate was tested according to GB/T 6670-2008. The hardness was tested according to GB/T 531-1999 with Shore O type hardness tester (SLX, Jiangdu Mingzhu Instrument Factory, Jiangdu, China). The tensile strength of the elongation at the break was tested according to GB/T 528-82, and the drawing speed for the measurement was 80 mm/min. The sound absorption properties of the samples were tested using standing wave tube (AWA1622, Hangzhou Aihua Instrument Co. LTD., Hangzhou, China) according to GB/T 18696.1-2004. The measurement was carried out in two sub wavebands of 90–1800 Hz and 800–6500 Hz by using a Φ96 mm × 1000 mm and a Φ30 mm × 350 mm standing-wave tubes, respectively. Samples for measuring the sound absorption coefficients in the working frequency of 100–6500 Hz were cut into two batches with Φ96 mm × 5 mm and Φ30 mm × 5 mm. Besides measuring the absorption coefficients at 125 Hz, 250 Hz, 500 Hz, 1000 Hz, 2000 Hz and 4000 Hz, according to GB/T 18696.1-2004, additional measurements are carried out at 375 Hz, 750 Hz, 1500 Hz, 3000 Hz and 5000 Hz, with the aim of further investigating the frequency dependent sound absorption performances for all samples. The measurement for each sample was repeated 5 times to calculate the average of all the data.

## 3. Results and Discussion

### 3.1. Microstructure Characteristics

**Figure 1:**
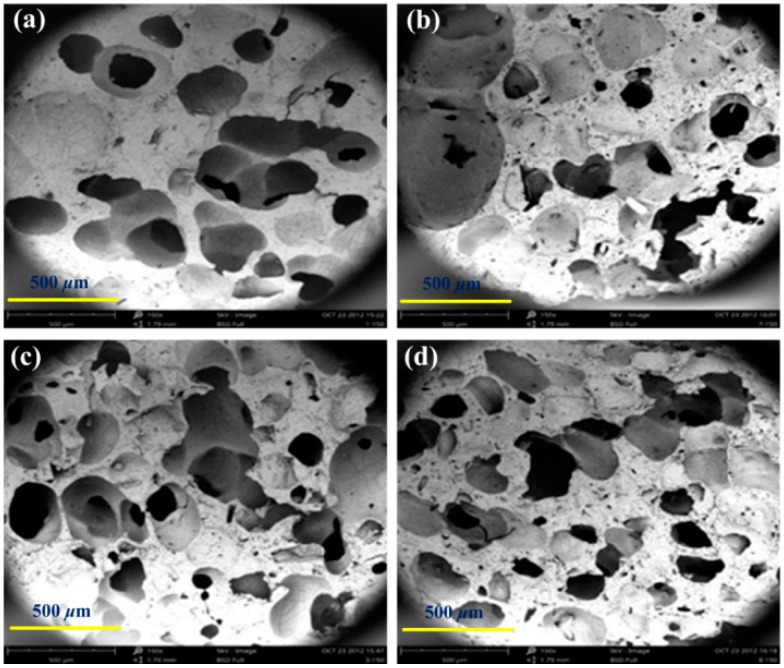
SEM photographs of silicone rubber foam fabricated with different additive amounts of NaCl. (**a**) 20 phr; (**b**) 40 phr; (**c**) 60 phr; and (**d**) 80 phr.

It can be seen from the SEM photographs shown in Figure 1 that the production rate and inner perforation of pores in foam samples changes significantly with the increasing amount of pore-foaming agents NaCl. With the small amount of NaCl (less than 20 phr), surface pores are small individual pores with an average size of approximately 100 μm. This is because the particle diameters of NaCl added into the silicon rubber were about 100 μm, and all these particles were dispersed in the colloid in the form of discrete particles after physical blending, leading to small and separated pores encapsulated by colloids after dissolve-separating foaming. With the increased added amount of NaCl to 40 phr, pores on the sample surface became larger (about 200 μm), and the inter-connected pores were partially formed, though most of the pores are still concave structures with larger sizes and poor penetration. With the further increasing added amount of NaCl (60 phr), the size and number of pores are all enlarged with an enhanced penetration effect, but the pore diameter fluctuates greatly due to the agglomeration of the NaCl. When the addition amount of NaCl was 80 phr, the sample had the largest number of pores with uniform sizes (about 300 μm), and the penetration rate of the pores is enhanced, which is the result of increased pore-forming nuclei in the colloids. Further increasing the addition amount of NaCl into the silicon rubber would not lead to an increased structural improvement of the samples. With the increasing addition amount of NaCl particles, the pore size formed in the silicon rubber would be increased, due to the agglomeration effect, which could be used to control the size of the porosities. Therefore, the optimized pore structure of the silicone rubber foam could be prepared with 80 phr of NaCl in the preparation method.

**Figure 2:**
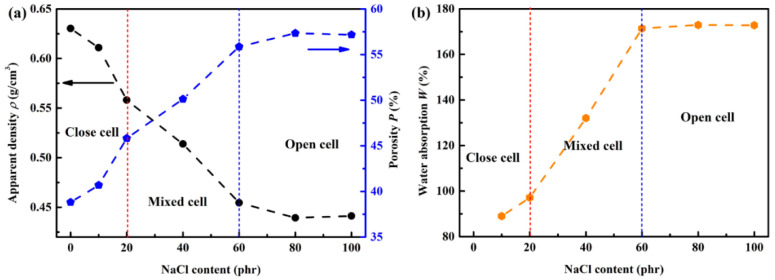
Cell structural properties of silicon rubber foams with different added amounts of NaCl. (**a**) Apparent density and porosity; (**b**) water absorption.

Previous studies have proved that the sound absorption efficiency of materials is highly correlated with the density, porosity, pore size, and other factors. Therefore, it is essential to investigate the structure-related properties of the fabricated foams. The measured results are plotted in Figure 2 as a function of the added amount of NaCl. As shown in Figure 2a, with the increasing added amounts of NaCl, the apparent density decreases first and then reaches to a constant (~0.44 g/cm^3^), while the porosity increases first and then reaches a constant (~57%). In the fabricating process of the silicon rubber foams, the filler NaCl particles dispersed in the colloid act as cavity nucleation sites that eventually grow into pores with the H_2_, which is released from the dehydrogenation and crosslinking reaction between the silicon-hydrogen bond (Si-H) of the hydrosilicone oil and the silanol (Si-OH) of the silicon rubber in the presence of Platinum catalyst. As shown in Figure 1, when the added amount of NaCl is less than 20 phr, pores inside the silicone rubber are mainly closed pores, and increased NaCl particles would result in perforation between pores due to the enlarged cavity nucleation sites density, leading to a mixed cell structure, as shown in Figure 1b. The filling amount of the NaCl into the silicon rubber would reach the saturated state at 60 phr, and further increasing the added amount of NaCl would not change the density and porosity anymore. Furthermore, the water absorption *W*, which is another important parameter for judging the type of pores in foams, is measured and plotted in Figure 2b. For closed-cell silicone rubber foam, individual pores could not have water absorption characteristics. Thus, the water absorption *W* is very low with small added amounts of NaCl, and gradually increases with increasing added amount of the pore forming agent NaCl since the number of the open cells was enlarged with the increased porosity of foams, as shown in Figure 2a. Analogously, further increasing the added amount of NaCl would not lead to the continuous enlargement of *W*, since the porosity and number of the open cells would reach their limits due to the saturation effect of NaCl in colloids.

### 3.2. Mechanical Properties

**Figure 3:**
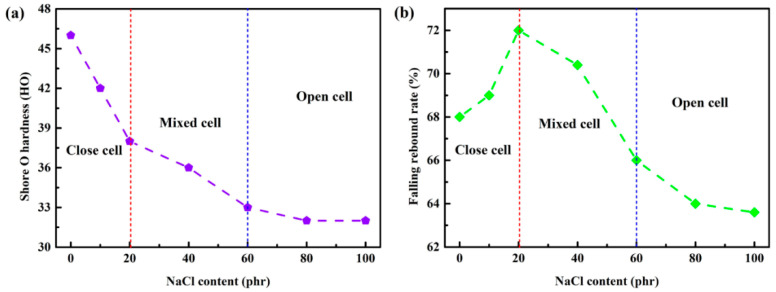
Elastic properties of silicon rubber foams with different added amount of NaCl. (**a**) Shore O hardness; (**b**) falling rebound rate.

It can be clearly concluded that the fabricated foams have complex microstructural geometries, and the structural characteristics show nonlinear relationships with the increasing added amount of the pore-forming agent NaCl. This complex cell microstructural geometry would directly affect the mechanical response of the silicon rubber foams. As shown in Figure 3a, with the increasing added amount of NaCl, the Shore O hardness of the samples gradually decay to a steady value of about 32 HO, which illustrates that the cell structure of the foams would significantly influence the hardness: small addition of NaCl (<20 phr) would form individual cells in the foam, leading to sharp decline of the hardness; with the increasing addition of NaCl (20–60 phr), the size and shape of cells would be changed gradually, and the reaction force was formed by the structural skeleton, which would decrease due to the enlarged aperture when silicone rubber is subjected to pressure, leading to the drop in hardness to a certain extent. As discussed in Figure 1, further increasing the addition of NaCl (>60 phr) would not lead to obvious changes in the pore structure inside the colloid; thus, the hardness of foams do not continue to decrease with a large addition of NaCl. In contrast, the elastic performance of the foams increases first and then gradually decreases with the increasing addition amount of NaCl, and a maximum falling rebound rate (70.4%) is achieved with 20 phr NaCl addition. This non-linear response should also be attributed to the cell structures of foams; closed and individual cells encased in colloids with small addition of NaCl can enhance the elastic properties, and non-uniform open cells in colloids would form a cavity inside the silicone rubber, which could reduce the elastic response, resulting in the falling rebound rate gradually decaying to a steady value of about 64%.

**Figure 4:**
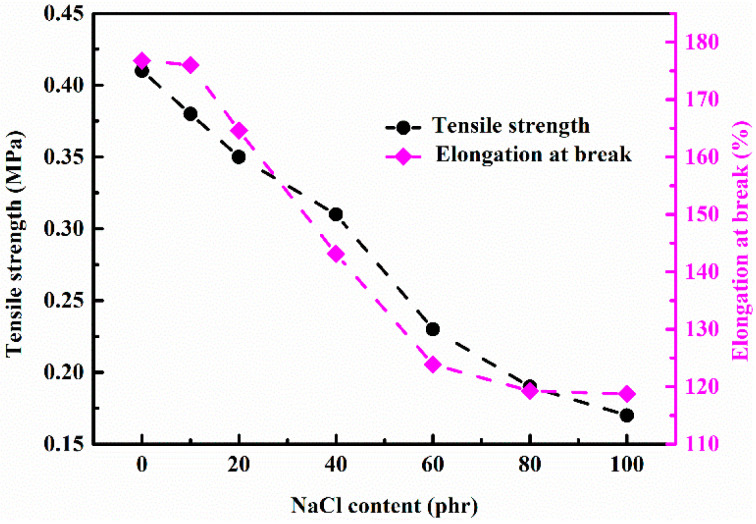
Effect of the addition amount of NaCl on the tensile strength and elongation at the break of silicon rubber forms.

Furthermore, the tensile strength and the corresponding elongation at the break of silicon rubber foams with different addition amounts of NaCl are plotted in Figure 4. With the increasing addition amount of NaCl, the tensile strength and the elongation at break all gradually decay to a steady value, respectively. When the added amount of NaCl is less than 10 phr, NaCl crystals are completely wrapped by the colloid, which would prevent the dissolution process, and these closed and individual pores would not cause significant cell structural defects; thus, the elongation at break does not change much. However, the tensile strength of the foams still shows significant reduction, which should be attributed to structural defects in the silicone rubber caused by the absence of physical or chemical bonds between NaCl and silicone rubber molecules, since NaCl does not have any free hydroxyl groups on the surface. When the addition amount of NaCl added is 10–60 phr, the tensile strength and elongation at break of the silicone rubber foams decrease at an approximately constant rate, which is the result of the linear increased open porosity of the silicone rubber foams. The effective area of the tensile force would decrease gradually, which would result in the stress concentration at the open cells, leading to the reduction of the mechanical properties. With further increasing of the addition amount of NaCl (>60 phr), the tensile strength and fracture of foams would not continuously decrease, since the porosity of silicon rubber foams (as shown in Figure 2a) tend to a fixed value, and slight changes of the tensile strength and elongation at break should be attributed to the random cell structures of foams. Therefore, the mechanical properties of silicon rubber foams would be reduced with the addition of NaCl, but the elastic property could be enhanced with a small amount of NaCl addition.

### 3.3. Formatting of Mathematical Components

**Figure 5:**
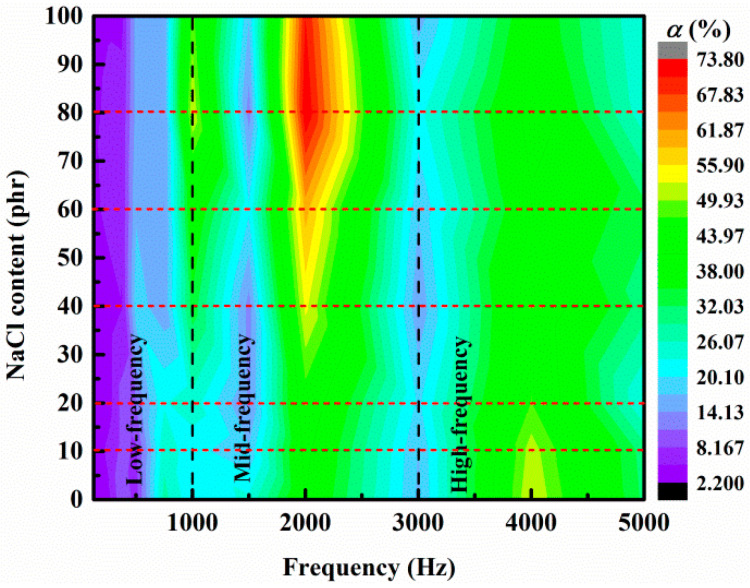
Frequency dependent sound absorption coefficient of silicon rubber foams with different added amounts of NaCl.

It has been well established that the sound absorption performance of the foams is closely related to their pore structure since the distribution of pathways in the foams have a great influence on the sound energy dissipation. The foams prepared with different added amounts of NaCl contain cavities and various structured pores (closed, partially open, and open pores), as shown in Figure 1; thus, the corresponding frequency dependent sound absorption coefficients were measured and plotted in Figure 5. The measurement was carried out for each sample at a frequency of 125 Hz, 250 Hz, 375 Hz, 500 Hz, 750 Hz, 1000 Hz, 1500 Hz, 2000 Hz, 3000 Hz, 4000 Hz and 5000 Hz, respectively. These experimental results were plotted as a contour map with interpolated values.

It can be seen from Figure 5 that, in the low frequency band (125–500 Hz), the silicone rubber hardly exhibits sound absorption performance, which should be the result of the wave-passing behavior caused by the scattering effect for long wavelengths. In the middle frequency range (1000–2000 Hz), the sound absorption coefficient of the silicone rubber is only 20.5% at 1000 Hz and 40.2% at 2000 Hz, while the silicon rubber foam prepared with 80 phr NaCl addition reaches 52.7% at 1000 Hz and 73.7% at 2000 Hz, indicating improved sound absorbing efficiency for middle frequency sound wave. In this frequency range, silicon rubber foams with open cell sound-absorbing structures could be equivalent to the Helmholtz resonator, since pores on the surface of silicon rubber foams, which were produced by the dissolution reaction to connect to the internal pores, formed necks with extremely small diameters, leading to the specific gas channel in the silicon rubber foams. The size and number of the internal pores increase with the increasing addition amount NaCl, leading to a decrease in the flow resistance of the foams. The harmonic vibrations of the curved wall were produced by the incident sound wave, which would result in the expansion and contraction of the air in internal pores, and the sound energy could be converted into heat energy due to the damping effect of the cavity wall on the air column, resulting in the consuming of sound energy. The middle frequency is closer to the natural frequency of silicone rubber, which would lead to a obvious resonance, and the air column in the tunnel moves has the highest vibration speed, resulting in the most effective energy conversion and the highest sound absorption coefficient in the middle frequency range. The effect of porosity has little effect on the sound absorption performance of foams at 3000 Hz, while the sound absorption coefficient of the open cell silicone rubber samples is lower than that of the closed-cell silicone rubber samples. The reason for this reduction in the sound absorption efficiency in the high frequency range should be attributed to the decreased flow resistance with high-energy sound waves. Sound waves transmitted from the neck to the inside of the foam are easier to pass through the other side through the internal pores, thereby reducing the sound absorption efficiency. On the contrary, silicon rubber samples without open cells have a good reflection effect to the high frequency sound wave. Therefore, the sound absorption performance of the silicon rubber foams consistently decreases with the increased addition amount of NaCl.

**Figure 6:**
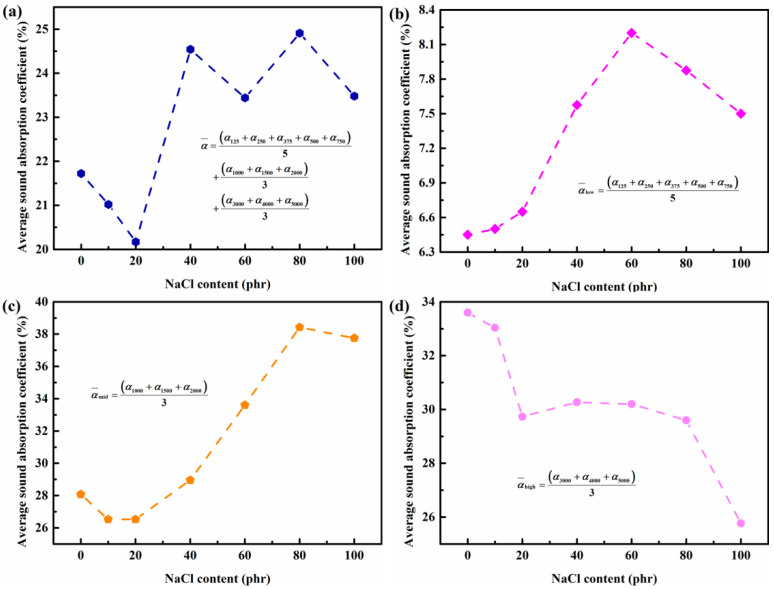
Effect of the addition amount of NaCl on sound absorption coefficient of silicon rubber foams in different frequency ranges. (**a**) 125–5000 Hz; (**b**) 125–750 Hz; (**c**) 1000–3000 Hz; (**d**) 3000–5000 Hz.

It is obvious that the sound absorption properties of the fabricated silicon rubber foams have a strong selective effect with respect to the frequency of incident sound wave; thus, the average sound absorption coefficients at different frequency ranges were calculated and plotted in Figure 6. In the whole frequency range, the average sound absorption coefficient $\bar{\alpha }$ of all samples are all larger than 0.2, and the addition of NaCl would reduce the $\bar{\alpha }$ of closed cell silicon rubber foams and significantly enlarge the $\bar{\alpha }$ of open cell silicon rubber foams. The addition amount of NaCl has no obvious effects on $\bar{\alpha }$, and the $\bar{\alpha }$ is mostly dependent on the cell strucutre of the foams. In the low-frequency range (<1000 Hz), the ${\bar{\alpha }}_{\mathrm{low}}$ for all samples are all very small due to the scattering effect for long wavelengths, but increasing the addition amount of NaCl could improve the sound absorption performance of the silicon rubber to a certain extent. In the high-frequency range (>2000 Hz), the ${\bar{\alpha }}_{\mathrm{high}}$ for all samples are decreased with increasing NaCl addition, and open cell silicon rubber foams show steady sound absorbing efficiency with different addition amounts of NaCl. The improved sound absorbing efficiency was mainly validated in the middle frequency (1000–2000 Hz), as shown in Figure 6c. With the small addition amount of NaCl (<20 phr), the ${\bar{\alpha }}_{\mathrm{mid}}$ of silicon rubber foams with closed cell structures is not much different from that of the dense silicon rubber without NaCl addition, since the flow resistance of the foams does not significantly enlarge with closed cell structures. Further increasing the NaCl addition to be higher than 20 phr, the ${\bar{\alpha }}_{\mathrm{mid}}$ will gradually increase to a steady value of about 38.4% with 80 NaCl added, which is 36.9% higher than that of the unopened silicone rubber sample. In this frequency range, the acoustics impedance of the foams are highly dependent on the size and number of the open cell in the silicone rubber; thereby, the increasing porosity of the foams would result in the enlarged penetration rate of the cells, which would decay the vibration effect of the air column generated by the sound energy in the cavity. When the amount of NaCl added is higher than 80 phr, the ${\bar{\alpha }}_{\mathrm{mid}}$ of the sample does not change much, since the open porosity of the sample has tended to be a relatively stable value, as shown in Figure 2a. Therefore, open cell silicon rubber foams are capable of middle sound wave absorption.

## 4. Conclusions

Silicon rubber foams, including NaCl as the pore forming particles, were fabricated via the dissolve-separating foaming method, with the aim of investigating the mechanical and sound absorption properties as a function of NaCl addition amount. Based on the obtained results, the following conclusions can be drawn:

1. The cell structures of the silicon rubber foams are highly dependent on the NaCl addition amount. A small amount of NaCl would result in the low porosity with closed cells, and a large addition amount of NaCl could cause increased connectivity of pores in the foams, which would result in different effects on the mechanical and sound absorption performances.
2. The mechanical properties of the fabricated foams, including hardness, falling rebound rate, tensile strength and elongation at break, decreases with increasing addition amounts of NaCl, indicating that the open cell silicon rubber foams easily process the various parts of complex shapes for various applications.
3. Open cell silicon rubber foams show improved sound absorption efficiency for the middle frequencies sound wave, which is attributed to the enhanced resonance matching caused by the open cell structures.

Therefore, the fabricated open cell silicon rubber foams show improved sound absorption performances for a sound wave of 1000–2000 Hz, and further investigation should focus on the cell structure manipulation, such as gradient cell structure design, to broaden the sound absorption bandwidth.

## Data Availability

Data sharing is not applicable to this article.

## References

[1] Tie T.S., Mo K.H., Putra A., Loo S.C., Alengaram U.J., Ling T.C. (2020). Sound absorption performance of modified concrete: A review. J. Build. Eng..

[2] Ning X., Qi J.Y., Wu C.L., Wang W.J. (2019). Reducing noise pollution by planning construction site layout via a multi-objective optimization model. J. Clean. Prod..

[3] Mikhailenko P., Piao Z.Y., Kakar M.R., Bueno M., Athari S., Pieren R., Heutschi K., Poulikakos L. (2020). Low-Noise pavement technologies and evaluation techniques: A literature review. Int. J. Pavement Eng..

[4] Bhingare N.H., Prakash S., Jatti V.S. (2019). A review on natural and waste material composite as acoustic material. Polym. Test..

[5] Tang X.N., Yan X. (2017). Acoustic energy absorption properties of fibrous materials: A review. Compos. Part A Appl. Sci. Manuf..

[6] Du Z.P., Yao D.X., Xia Y.F., Zuo K.H., Yin J.W., Liang H.Q., Zeng Y.P. (2020). The sound absorption properties of highly porous silicon nitride ceramic foams. J. Alloy. Compd..

[7] Kalauni K., Pawar S.J. (2019). A review on the taxonomy, factors associated with sound absorption and theoretical modeling of porous sound absorbing materials. J. Porous Mater..

[8] Wang J.Z., Ao Q.B., Ma J., Kang X.T., Wu C., Tang H.P., Song W.D. (2019). Sound absorption performance of porous metal fiber materials with different structures. Appl. Acoust..

[9] Ao Q.B., Wang J.Z., Ma J., Tang H.P. (2019). Effect of Pore Structure on the Sound Absorption Properties of the Stainless Steel Fiber Porous Materials. Rare Metal Mater. Eng..

[10] Ao Q.B., Wang J.Z., Tang H.P., Zhi H., Ma J., Bao T.F. (2015). Sound Absorption Characteristics and Structure Optimization of Porous Metal Fibrous Materials. Rare Metal Mater. Eng..

[11] Liu S.T., Chen W.J., Zhang Y.C. (2014). Design optimization of porous fibrous material for maximizing absorption of sounds under set frequency bands. Appl. Acoust..

[12] Cao L.T., Fu Q.X., Si Y., Ding B., Yu J.Y. (2018). Porous materials for sound absorption. Compos. Commun..

[13] Bowen C.R., Perry A., Lewis A.C.F., Kara H. (2004). Processing and properties of porous piezoelectric materials with high hydrostatic figures of merit. J. Eur. Ceram. Soc..

[14] Soltani P., Taban E., Faridan M., Samaei S.E., Amininasab S. (2020). Experimental and computational investigation of sound absorption performance of sustainable porous material: Yucca Gloriosa fiber. Appl. Acoust..

[15] Yu T.M., Jiang F.C., Wang J.H., Wang Z.Q., Chang Y.P., Guo C.H. (2020). Acoustic insulation and absorption mechanism of metallic hollow spheres composites with different polymer matrix. Compos. Struct..

[16] Gao N.S., Hou H. (2017). Low frequency acoustic properties of a honeycomb-silicone rubber acoustic metamaterial. Mod. Phys. Lett. B.

[17] Kumar A., Mollah A.A., Keshri A.K., Kumar M., Singh K., Rallabhandi K.D.V.S., Seelaboyina R. (2016). Development of Macroporous Silicone Rubber for Acoustic Applications. Ind. Eng. Chem. Res..

[18] Gao N.S., Lu K. (2020). An underwater metamaterial for broadband acoustic absorption at low frequency. Appl. Acoust..

[19] Vaquette C., Frochot C., Rahouadj R., Wang X. (2008). An innovative method to obtain porous PLLA scaffolds with highly spherical and interconnected pores. J. Biomed. Mater. Res. Part B.

